# 39 Implementation of a Screening Tool for Wellbeing in the Burn Clinic for Patients < 5 Years

**DOI:** 10.1093/jbcr/irac012.042

**Published:** 2022-03-23

**Authors:** Erica Y Magnuson, Victor C Joe, Theresa L Chin

**Affiliations:** UCI Health, Orange, California; UCI Health Regional Burn Center, Orange, California; University of California Irvine, Orange, California

## Abstract

**Introduction:**

Pediatric burn survivors and their parents experience higher rates of psychological disorders compared to their uninjured peers. In the early years, children experience rapidly changing development, which can impact responses to psychological outcomes. Early identification of developmental delays is vital to recovery and coping with burn injury. As a quality improvement project, a survey tool was implemented in the burn clinic to screen for issues impacting pediatric burn survivors and their family’s coping, who may benefit from additional referrals.

**Methods:**

A free comprehensive tool, which screens for behavior, development and family stressors was chosen based on its high reliability, validity, and usability including being available in multiple languages. Prior to implementation and following the trial phase, staff completed qualitative and quantitative surveys regarding their input on the importance of screening and ease of execution. The Child Life Specialist (CLS) screened and scored children ages 12 to 65 months upon their initial visit and were rescreened two to four months later if they scored “Needs Review” in any category, indicating additional assessment was needed. Those who scored 50% of expected score for their age on initial assessment or a repeat “Needs Review” on the second assessment, were referred to additional resources by the CLS, social worker or physician team.

**Results:**

Of the 43 children whose parents completed the survey (3 in Spanish), there were 25 males and 18 females. The median age was 2.6 years (IQR:2.0-3.52). The score of the survey determined that 42% (n=18) of children evaluated as “Needs Review” or ≤50% expected in one or more category on initial assessment. Only 5 children were already receiving services and two children scored ≤50% threshold score for immediate referral. Initial surveys demonstrated staff (n=9) strongly agreed assessing children’s need for support services was important. Majority (88%) of children were assessed upon their initial visit to the outpatient burn clinic. Most staff agreed the tool had been effectively implemented in the outpatient clinic.

**Conclusions:**

The tool was effectively integrated into the burn clinic and identified children who needed referral for support services. Screening in the burn clinic provides an opportunity to identify critical factors related to children’s development and behavior, which can compound psychological responses to burn injury and addresses parental concerns for development.

